# A nucleotide‐independent, pan‐RAS‐targeted DARPin elicits anti‐tumor activity in a multimodal manner

**DOI:** 10.1002/1878-0261.70061

**Published:** 2025-06-15

**Authors:** Jonas N. Kapp, Wouter P. R. Verdurmen, Jonas V. Schaefer, Kari Kopra, Gabriela Nagy‐Davidescu, Elodie Richard, Marie‐Julie Nokin, Patrick Ernst, Rastislav Tamaskovic, Martin Schwill, Ralph Degen, Claudia Scholl, David Santamaria, Andreas Plückthun

**Affiliations:** ^1^ Department of Biochemistry University of Zurich Switzerland; ^2^ Department of Chemistry University of Turku Finland; ^3^ Bordeaux Institute of Oncology (BRIC), INSERM U1312 University of Bordeaux France; ^4^ Laboratory of Biology of Tumor and Development (LBTD), GIGA‐Cancer University of Liege Belgium; ^5^ ACTION Laboratory, IECB, INSERM U1218 University of Bordeaux France; ^6^ Division of Applied Functional Genomics German Cancer Research Center (DKFZ) Heidelberg Germany; ^7^ National Center for Tumor Diseases (NCT) Heidelberg Germany; ^8^ Centro de Investigación del Cáncer CSIC‐Universidad de Salamanca Spain

**Keywords:** cancer, Designed Ankyrin Repeat Protein, drug development, oncogene, RAS, small GTPase

## Abstract

The KRAS oncoprotein is a frequent tumor driver in lung, pancreatic, and colorectal cancers and has proven to be a challenging pharmaceutical target. The first KRAS‐targeted therapeutics are now being tested in clinical trials but the consequences of preferentially targeting the GDP or GTP state of KRAS and the relevance of RAS nanoclustering have remained unclear. Here we report a Designed Ankyrin Repeat Protein (DARPin) that recognizes the RAS switch I/II region with low nm affinity, independently of the nucleotide bound (GDP‐ or GTP state). This DARPin, termed ‘784_F5’, occupies the effector recognition lobe, resulting in interference with SOS‐mediated activation, RAS downstream effector interactions, and KRAS nanoclustering. Consequently, this anti‐RAS DARPin potently blocks downstream signaling, leading to a strong reduction in proliferation and anchorage‐independent growth in RAS‐dependent cell lines. We showed that the expression of ‘784_F5’, the pan‐RAS, nucleotide‐independent DARPin can lead to tumor regression in a colorectal xenograft model which may hold promise for further investigation and development.

## Graphical abstract

We report a Designed Ankyrin Repeat Protein that binds and inhibits RAS proteins, which serve as central cell signaling hubs and are essential for the progression of many cancers. Its unique feature is that it does not discriminate between different RAS isoforms or mutations and is capable of binding to RAS in both its active (GTP‐bound) and inactive (GDP‐bound) states.

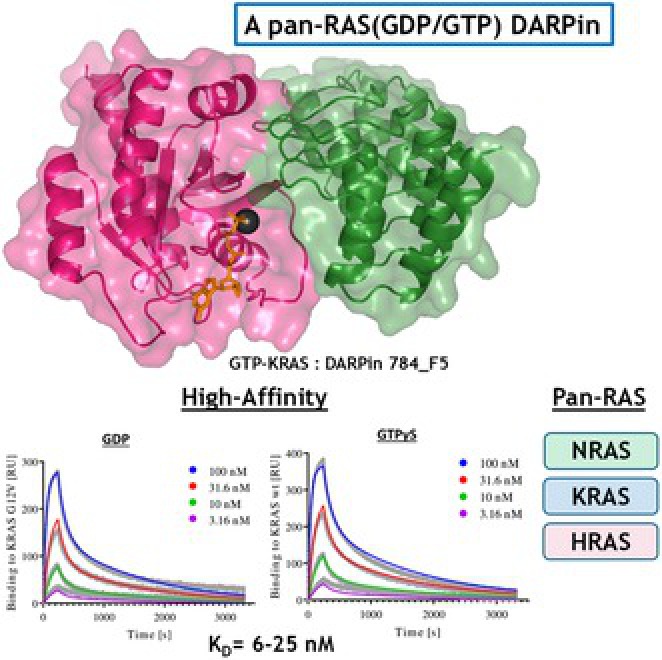

**Abbreviations**
AKT: protein kinase B (AKR mouse strain thymoma)
BCA: bicinchoninic acid assay
BRET: bioluminescence resonance energy transfer
CMV: cytomegalovirus
DARPin: Designed Ankyrin Repeat Protein
DMEM: Dulbecco's Modified Eagle Medium
DTT: dithiothreitol
EDTA: ethylenediaminetetraacetic acid
ELISA: enzyme‐linked immunosorbent assay
FCS: fetal calf serum
GDP: guanosine diphosphate
GMP‐PNP: 5′‐guanosine‐β,γ‐imidodiphosphate
GTP: guanosine‐5′‐triphosphate
GTPγS: guanosine 5′‐O‐(3‐thio)triphosphate
H&E: Hematoxylin & Eosin staining
HPLC: high‐performance liquid chromatography
HRAS: Harvey rat sarcoma viral oncogene homolog
HTRF: homogeneous time‐resolved fluorescence
IMAC: immobilized metal‐ion affinity chromatography
IP: immunoprecipitation
IPTG: isopropyl β‐d‐thiogalactopyranoside
K_D_
: equilibrium dissociation constant
KRAS: Kirsten rat sarcoma viral oncogene homolog
LNPs: lipid nanoparticles
mAb: monoclonal antibody
MAPK/ERK: mitogen‐activated protein kinase/extracellular signal‐regulated kinase
mTOR: mammalian target of rapamycin
MWCO: molecular weight cut‐off
NHS ester: *N*‐hydroxysuccinimide ester
NMR: nuclear magnetic resonance
NRAS: neuroblastoma RAS viral oncogene homolog
NSG mice: NOD scid gamma mice
PBS: phosphate‐buffered saline
PDB: Protein Data Bank
PI3K: phosphatidylinositol‐3‐kinase
PLC: phospholipase C
QRET: quenching resonance energy transfer
RAF: rapidly accelerated fibrosarcoma
RALGDS: Ral guanine nucleotide dissociation stimulator
RBD: RAS‐binding domain
RPMI 1640: Roswell Park Memorial Institute 1640 medium
SA: Streptavidin
scFv: single‐chain variable fragment of an antibody
SDS/PAGE: sodium dodecyl‐sulfate polyacrylamide gel electrophoresis
SOS: son of sevenless
SPR: surface plasmon resonance
TEV: Tobacco Etch Virus
TR‐FRET: time‐resolved Förster resonance energy transfer
w/v: weight/volume

## 1 Introduction

The RAS family of small GTPases, including KRAS, NRAS, and HRAS, act as molecular switches that control a variety of intracellular signaling processes. The oncogenic nature of mutated members of the Ras family has been recognized for over 25 years [1]. Mutations in RAS proteins are found in about 19–23% of human cancers and are often considered driver mutations [2, 3, 4]. Mutations in KRAS are especially prevalent, occurring in up to 90% of pancreatic, 50% of colon, and 25% of lung adenocarcinomas, and are frequently associated with poor prognoses [5, 6]. In general, the continuous activation of the molecular switch RAS, caused by the loss of GTPase activity, leads to the permanent initiation of downstream signaling, resulting in uncontrolled cell proliferation. RAS proteins can activate various downstream effectors including a set of kinases and nucleotide exchange factors, such as PI3Ks, RALGDS, RAF, and PLC [7]. Among the downstream signaling components of RAS‐mediated oncogenesis, the MAPK/ERK and PI3K‐AKT pathways are considered the most important, significantly contributing to cell proliferation, survival, tissue invasion, and metastasis [8, 9].

Despite intense efforts and a recent revival of interest in targeting RAS family members, treatment options for patients with RAS‐driven cancers remain severely limited [10]. The direct targeting of RAS proteins, especially mutant forms of KRAS, has been historically challenging due to the protein's high affinity for GTP and the lack of suitable small‐molecule binding pockets. This has rendered KRAS one of the most notorious ‘undruggable’ targets in cancer therapy. However, the discovery of the switch II pocket (SII‐P) by the Shokat laboratory marked a significant breakthrough, enabling the development of covalent inhibitors that specifically target the G12C mutant form of KRAS by irreversibly binding to the mutant cysteine residue [11, 12]. In recent years, further advancements have been made in the noncovalent targeting of other KRAS mutations, notably the G12D mutation [13, 14]. Moreover, the development of a pan‐KRAS and a multi‐RAS(ON) inhibitor represents additional innovative approaches in the RAS‐targeting landscape [15, 16].

RAS binding and subsequent interference with RAS function can also be accomplished through exogenous proteins. While the idea of targeting RAS with biologics was established early on [17], significant progress in the development of well‐characterized RAS binders has been made within the last 20 years. Available protein‐derived RAS inhibitors vary in their molecular scaffolds, specificities in terms of RAS isoform and mutants, as well as their ability to recognize different nucleotide‐bound states of RAS. Tomazini and Shifman recently summarized the vast collection of RAS biologics described to date [18]. Further approaches have been reported in which RAS binders have been combined with E3‐ligase domains to induce proteasomal degradation [19, 20], or where RAS‐cleaving proteases have been utilized [21] or dominant negative RAS effectors have been engineered [22, 23].

However, the therapeutic use of proteins as intracellular inhibitors is currently limited by delivery technologies. Nonetheless, these proteins can serve as blueprints for the development of small‐molecule or peptide drugs, and protein and gene delivery technologies are in continuous further development [24, 25]. An ideal starting point for probing important features of such a drug would be a collection of inhibitors based on the same scaffold that vary in their epitope and/or specificity for (K)RAS. While such a set is nearly complete for the class of monobodies, the class of Designed Ankyrin Repeat Proteins (DARPins) is rather incomplete [19, 26, 27, 28, 29]. To date, GDP‐ and GTP‐specific pan‐RAS DARPins have been described for the effector lobe [30]. Additionally, a KRAS‐specific, nucleotide‐state agnostic DARPin was reported to recognize the allosteric lobe [31].

DARPins are highly stable binding proteins that can be selected against most targets, providing a continuous, concave binding interface [32]. With a size smaller than 20 kDa and no disulfide bridges, they retain their conformation and activity in the cytosol without the need for a stabilizing fusion protein [33, 34]. This allows for a more direct characterization of the binder's effects without the need to consider additional steric contributions of the fusion protein, as is necessary in the case of monobodies [26, 27, 28, 29].

We previously mentioned RAS‐binding DARPins that either compete with RAF for RAS binding (termed RAS0104 and RAS0107) or do not compete (RAS0109). These DARPins were used as tools to confirm the role of RAS in the compensatory activation of the PI3K/AKT pathway in BT474 cells, subverting HER2 inhibition [35]. In this study, we present an extensive characterization of a DARPin (RAS0107, renamed to 784_F5) that binds tightly to all isoforms of RAS, both in their GTPγS‐loaded active state and in their GDP‐loaded inactive state. We provide a biochemical and functional characterization of this DARPin, aiming to link mechanisms of RAS inhibition to their consequences on proliferation, RAS downstream signaling, anchorage‐independent growth, and *in vivo* growth of RAS‐dependent cell lines.

## 2 Materials and methods

### 2.1 Ribosome display and DARPin screening

Ribosome display selections against KRAS Q61H were performed essentially as described previously, though in a 96‐well format wherever possible [36]. The targets, containing the residues 1–169, were expressed with a C‐terminal Avi‐tag. *E. coli* BL21 (DE3) cells were co‐transformed with an *E. coli* biotin ligase BirA expression plasmid for *in vivo* biotinylation.

The fully synthetic library consists of N3C‐DARPins with three randomized internal repeats, containing a mixture of non‐randomized and randomized N‐terminal and C‐terminal capping repeats [32, 37]. Selections were performed over four rounds with decreasing concentrations of biotinylated target protein for the first three cycles, an off‐rate selection using non‐biotinylated target protein in the third cycle followed by a fourth round with less stringent conditions [36, 38]. The final enriched pool of the DARPin‐encoding cDNA was cloned as fusions with an N‐terminal MRGSHis_6_‐ and C‐terminal FLAG‐tag into a derivative of pQE30 (QIAGEN, Hilden, Germany) containing a *lacI* ^*q*^ gene via unique BamHI and HindIII restriction sites under the control of a T5lac promoter.

After transformation of *E. coli*, 800 single DARPin clones per selection were expressed in 96‐well format and lysed by addition of a concentrated Tris/HCl‐based lysis buffer containing n‐octyl β‐d‐thioglucopyranoside (OTG), lysozyme, and universal nuclease. Crude extract ELISAs and subsequent sequencing to identify unique clones yielded in total 94 unique DARPins against both clones (65 against KRAS Q61H, 29 against NRAS wt). Positive hits were further analyzed for binding by homogeneous time‐resolved fluorescence (HTRF) via their 6xHis and FLAG‐tags. For this purpose, binding of the FLAG‐tagged DARPins to streptavidin‐immobilized biotinylated target protein was measured using HTRF (donor: Streptavidin‐Tb cryptate (610SATLB, Cisbio, Codolet, France), acceptor: mAb anti‐FLAG M2‐d2 (61FG2DLB, Cisbio)). Experiments were performed at room temperature in white 384‐well Optiplate plates (PerkinElmer, Waltham, MA, USA) using the Taglite assay buffer (Cisbio) at a final volume of 20 μL per well.

Single clones were expressed on a small scale and purified using a 96‐well IMAC column (HisPur™ Cobalt plates, Thermo Scientific, Waltham, MA, USA). DARPins after IMAC purification were analyzed for potential oligomerization tendency at a concentration of 10 μm on a Superdex 200 increase 5/150 GL column (GE Healthcare, Chicago, IL, USA) using an LC1200 HPLC system (Agilent, Santa Clara, CA, USA) with PBS containing 400 mm NaCl as the running buffer. Absorbance at 280 nm was recorded.

### 2.2 Protein expression

DARPins were expressed as described before [39, 40, 41]. All expression constructs are shown in Table S1. Biotinylated RAS proteins carrying an Avi‐tag were expressed in *E. coli* AVB100 (Avidity). Expression was carried out for 16 h at 18 °C in Terrific Broth containing 2% (v/v) EtOH. Expression of KRAS was induced with 0.2 mm IPTG after the culture had reached OD600 = 0.8. All steps of protein purification were performed at 4 °C. Bacterial pellets were resuspended in lysis buffer (50 mm Tris/HCl pH 8.0, 10% (w/v) glycerol, 300 mm NaCl, 20 mm imidazole; 10 mm MgCl_2_, 0.1 mm GDP containing 25 ng·mL^−1^ DNAse, 1 mg·mL^−1^ lysozyme, and the protease inhibitors Leupeptin, Petabloc SC, and Pepstatin‐A and lysed via sonication). After clarification, RAS proteins were purified using Ni‐NTA immobilized metal‐ion affinity chromatography (IMAC) and dialyzed to 50 mm HEPES pH 7.4, 10% (w/v) glycerol, 1 mm DTT, 150 mm NaCl, 20 mm imidazole; 10 mm MgCl_2_ involving TEV‐cleavage where needed. TEV protease and uncleaved protein were removed by reverse IMAC and further purified by size exclusion chromatography to provide a monomeric protein.

### 2.3 Loading of RAS with nucleotides

To load Ras variants with the nucleotides GTPγS, GMP‐PNP, or GDP, EDTA was added to 10 mm, and the nucleotide was added to a final concentration of 2 mm. The mixtures were incubated for 15 min at 30 °C, after which MgCl_2_ was added to 64 mm to stop the exchange reaction. Buffer exchange into the desired buffer for the experiment was accomplished using 7 kDa MWCO ZEBA Spin columns (Thermo Scientific) and an overnight dialysis.

### 2.4 Surface plasmon resonance

A ProteOn XPR36 (Bio‐Rad, Hercules, CA, USA) was used for all surface plasmon resonance (SPR) experiments. RAS variants, biotinylated via an Avi‐tag, were activated with the desired nucleotide as described above and immobilized on neutravidin‐coated sensor chips (ProteOn NLC, Bio‐Rad) at a density of about 2.5–5.0 ng·μL^−1^ in running buffer (PBS, 0.005% Tween‐20, 5 mm MgCl_2_, 10 μm GTPɤS). DARPins were run over the chip in duplicate at the concentrations 3.16, 10, 31.6, and 100 nm. For the analysis, a two‐state induced fit model was used from the proteon manager Software version 3.1.0.6 (Bio‐Rad). In this model, the DARPin (*A*) and Ras (*B*) form an initial complex *AB*, which then rearranges into a final complex (*AB*)* (E).

(1) $$A+B\overset{k_{\mathrm{on}}}{\underset{k_{\mathrm{off}}}{⇌}}\mathrm{AB}\overset{k_{2}}{\underset{k_{-2}}{⇌}}{\left(\mathrm{AB}\right)}^{*}$$

The equilibrium constants of the individual steps are then described by Eqn (2)

(2) $$K_{D1}=\frac{k_{\mathrm{off}}}{k_{\mathrm{on}}}\;\text{and}\;K_{2}=\frac{k_{-2}}{k_{2}}$$

### 2.5 Molecular cloning

For the generation of doxycycline‐inducible cell lines, DARPins 784_F5 and the control DARPin E3_5 were first subcloned, including a N‐terminal MRGS‐6xHis‐ and a C‐terminal FLAG‐tag from the pQIq backbone into the pDONR221 (Invitrogen, Carlsbad, CA, USA) shuttle vector via Gateway cloning using the following primers:

- 5′‐GGGGACAAGTTTGTACAAAAAAGCAGGCTTCACCATGGGCATGAGAGGATCGCATCACCAT‐3′
- 5′‐GGGGACCACTTTGTACAAGAAAGCTGGGTTTTACTTGTCGTCGTCATCCTTGTAGTC‐3′.

Doxycycline‐inducible expression vectors were then generated by subcloning the DARPins into the Gateway vector pLenti CMV/TO Puro DEST.

To monitor KRAS nanoclustering in a BRET assay, N‐terminal fusions of KRAS to either mNeonGreen or NanoLuc were constructed in a pcDNA3.1(+) vector (Invitrogen). KRAS point mutations were introduced either by site‐directed mutagenesis (QuikChange, Agilent) or by replacing the KRAS cDNA with a synthetic DNA string (GeneArt, Regensburg, Germany) carrying the desired mutation. Similarly, DARPins were cloned as N‐terminal fusions to mNeonGreen to assess in‐cell target engagement via BRET. All sequences were confirmed by Sanger sequencing. DARPins or the NS1 monobody [28] used as inhibitors in the KRAS nanoclustering assay were cloned into a pcDNA3.1(+) vector as well.

The plasmid pLenti CMV/TO Puro DEST (670‐1) was a gift from Eric Campeau and Paul Kaufman (Addgene plasmid # 17293, Watertown, MA, USA) [42].

### 2.6 Crystallization

To generate a complex, GMP‐PNP‐loaded KRAS(1–186) and DARPin 784_F5 were mixed in equimolar amounts and incubated for 1 h at 4 °C. The mixture was subsequently applied to preparative size exclusion chromatography in order to isolate the complex. Complexed proteins were then concentrated to 8.8 mg·mL^−1^ and set up for crystallization in sitting‐drop vapor‐diffusion experiments in 96‐well plates. Three different ratios of reservoir : protein solution (1 : 1, 2 : 1, and 3 : 1) in 300 nL drops were used per well and were incubated against 75 μL reservoir solution at 4 °C. Crystals grew within 25 days in 0.2 m potassium sodium tartrate, 20% w/v PEG 3350.

The crystals were mounted in cryo‐loops from Hampton Research and flash‐cooled in liquid nitrogen with ethylene glycol as further cryoprotectant. X‐ray diffraction data were collected at a wavelength of 1.0 Å on beamline X06SA at the Swiss Light Source, Paul Scherrer Institute (PSI), Villigen, Switzerland, equipped with an EIGER 16M detector (Dectris, Baden‐Wattwil, Switzerland). The data were processed with xds, xscale, and xdsconv [43]. Structures were solved by molecular replacement with phaser [44] using structures 4YDW and 5F2E as reference models. Calculation of the electron density and refinement were performed using phenix.refine [45]. For model building and preparation of figures, we used phenix.autobuild, phenix.refine, coot, and pymol [46, 47, 48]. Protein:protein interactions were analyzed with the help of pdbepisa and ligplot software [49, 50].

### 2.7 Co‐immunoprecipitation

HEK293T cells were transfected with HA‐tagged HRAS, NRAS, or KRAS4B in a pcDNA3.1 backbone using TransIT®‐LT1 (Mirus, Madison, WI, USA). Cells were washed and lysed after 60 h in IP buffer (50 mm Tris/HCl pH 7.4, 150 mm NaCl, 1% NP‐40, 5% glycerol, 10 mm MgCl_2_, 50 units·mL^−1^ Benzonase). Total protein concentration was determined via BCA protein assays and normalized for 500 μg protein input. DARPins were immobilized on magnetic anti‐FLAG beads (Pierce, Rockford, IL, USA) and used to co‐immunoprecipitate RAS from cell lysates. DARPin‐RAS complexes were eluted by 3x DYKDDDDK peptide (Pierce) and visualized via western blotting, staining the representative HA‐ and FLAG‐tags.

### 2.8 Cell culture

All cell lines were maintained at 37 °C with 5% CO_2_ and regularly tested for mycoplasma. Cells were grown in DMEM supplemented with 10% fetal calf serum (FCS) and 1% penicillin/streptomycin. All cell lines have been authenticated in the past 3 years via single nucleotide polymorphism (SNP)‐profiling.

### 2.9 BRET assay

HEK293T cells were seeded in white 96‐well clear bottom plates (Corning) 24 h pre‐transfection. Co‐transfections of the reporter plasmids were carried out with TransIT‐293 (Mirus) using the manufacturer's suggested protocol. 24 h post‐transfection, the medium was exchanged for DMEM without phenol red (Life Technologies, Carlsbad, CA, USA) containing 4% FCS. 48 h post‐transfection, BRET measurements were taken at a Victor 3 Multilabel Plate Reader after the addition of 10 μL 32 μm coelenterazine 400a (Cayman Chemical, Ann Arbor, MI, USA), resulting in a 2.9 μm final concentration. Emission of mNeonGreen and NanoLuc was observed for 2 s at 535 ± 25 and 460 ± 25 nm, respectively. Expression of both reporters was monitored by measuring mNeonGreen (Ex 485 nm, Em 535 nm) before the addition of luciferase substrate and measuring total luminescence directly after the BRET measurements for 0.3 s.

BRET ratios were calculated based on the following formula for each transfected well:

$$\text{BRET Ratio}=\frac{{\mathrm{Em}}_{535\;\mathrm{nm}}}{{\mathrm{Em}}_{460\;\mathrm{nm}}}-\mathrm{Cf}$$

$$\mathrm{Cf}=\frac{{\mathrm{Em}}_{535\;\mathrm{nm}}}{{\mathrm{Em}}_{460\;\mathrm{nm}}}\;\left(\text{Donor only sample}\right)$$

### 2.10 Generation of inducible cell lines

Cells were sequentially transduced with lentiviral plasmids carrying the TetR tetracycline repressor protein (pLenti TetR Blast) and those carrying the DARPins E3_5 or 784_F5 (pLenti CMV/TO Puro DEST), respectively. Lentiviral particles were produced by co‐transfection of HEK293T cells with the pLenti expression vectors, packaging plasmid psPAX2, and envelope plasmid pMD2.G using TransIT®‐LT1, and virus was harvested after 48 and 72 h. Cells were incubated with lentiviral supernatants in the presence of 8 μg·mL^−1^ polybrene for 30 h, and infected cells were selected with 5 μg·mL^−1^ blasticidin (TetR) for 14 days or 3 μg·mL^−1^ puromycin for 5 days.

The plasmids pMD2.G and psPAX2 were a gift from Didier Trono (Addgene plasmid #12259 and #12260). The plasmid pLenti TetR Blast (716‐1) was a gift from Eric Campeau and Paul Kaufman (Addgene plasmid #17492) [42].

### 2.11 Proliferation and colony formation

Cell lines were plated in 96‐well plates for proliferation assays (1000–4000 cells per well) or in 6‐well plates for colony formation assays with or without 1 μg·mL^−1^ doxycycline to induce DARPin expression.

Proliferation was either monitored for four consecutive days via CellTiter 96 AQueous One Solution Cell Proliferation Assay (Promega, Madison, WI, USA) and values were normalized to day one (HEK293T) or measured after 72 h with CellTiter‐Blue (Promega).

For the colony formation assay, 1000 cells were seeded as single cells in 3 mL of complete medium and allowed to form colonies for 10 days, while exchanging media every 72 h. Cells were then washed with PBS, fixed for 10 min with ice‐cold methanol, and subsequently stained with a 2.5% (w/v) crystal violet solution (Sigma‐Aldrich, Burlingtin, MA, USA) for 20 min. After removing excess stain, plates were washed thoroughly with water and dried overnight. The crystal violet stained plates were imaged, and the covered area was analyzed with the ImageJ plugin ColonyArea [51].

### 2.12 Anchorage‐independent growth

Soft agar assays were performed in 12‐well plates. 10 000 cells were seeded in RPMI 1640 containing 10% FCS and 0.35% noble agar with or without 1 μg·mL^−1^ doxycycline and plated on top of a layer of RPMI 1640 containing 10% FCS and 0.5% noble agar. 200 μL of medium was applied on day 3 after seeding and exchanged every 72 h. After 10 days, colonies were stained with 0.5% (w/v) crystal violet and photographed with a Zeiss Cell Observer. Images were processed using the zeiss axiovision software.

### 2.13 Western blotting

Cells were lysed in RIPA buffer containing 1× Halt protease inhibitor cocktail (Pierce) and 1× Halt phosphatase inhibitor cocktail (Pierce). All primary antibodies were obtained from Cell Signaling Technology (Danvers, MA, USA) unless otherwise noted: phospho‐AKT S473 (#9271S), total AKT (#2967), phospho‐MEK1/2 (#9154S), total MEK1/2 (#4694S), phospho‐ERK1/2 (#4370), total ERK (#9102), phospho‐p90RSK S380 (11989S), Anti‐FLAG tag (#2368). 50 μg of total protein was resolved by SDS/PAGE, transferred to a nitrocellulose membrane, and blocked in Odyssey blocking buffer. Immunodetection was performed with the indicated primary antibodies, followed by secondary goat, anti‐rabbit, and anti‐mouse antibodies labeled with CF680 or CF790 (Biotium, Fremont, CA, USA), and imaging was performed using the Odyssey CLx instrument (LI‐COR Biosciences, Lincoln, NE, USA).

### 2.14 Alexa647‐GDP, Alexa647‐GTP, and Eu(III)‐GTP labeling

Nonadentate Eu(III)‐chelate conjugation to GDP was performed as described previously with GTP [52, 53]. Conjugations with Alexa Fluor 647 NHS ester were performed in a similar manner in 50 mm carbonate buffer pH 8.3 [54]. Briefly, the label was conjugated to either 2′/3′‐(6‐aminohexylcarbamoyl)‐GTP (2′/3′‐AHC‐GTP) or 2′/3′‐AHC‐GDP (BIOLOG Life Science Institute, Bremen, Germany) in 50 mm carbonate buffer, pH 9.8, using a 1 : 3 molar ratio of nucleotide/label in a total reaction volume of 150 μL. Conjugation reactions were protected from light and incubated for 18 h at room temperature with slow shaking. {2,2′,2″,2‴‐{[4′‐(4‴‐isothiocyanatophenyl)‐2,2′,6′,2″‐terpyridine‐6,6″‐diyl]bis(methylenenitrilo)}tetrakis(acetate)}europium(III) (Nonadentate Eu(III)‐chelate) was purchased from QRET Technologies (Turku, Finland). Alexa Fluor 680 NHS (A37567) and Alexa Fluor 647 NHS (A37573) were purchased from Thermo Fisher Scientific.

### 2.15 Raf‐RBD‐Alexa680 and Eu(III)‐streptavidin (SA) labeling

Alexa Fluor 680 NHS ester was used in conjugations of the RAS‐binding domain of Raf (Raf‐RBD) to prepare Raf‐RBD‐Alexa680 as previously described [53]. Raf‐RBD (0.1 mg) was labeled with a two‐fold molar excess of Alexa680 in 175 μL in 50 mm carbonate buffer pH 8.3 for 30 min while protected from light. Alexa680‐RBD conjugates were separated from the free Alexa680 label using NAP‐5 columns (GE Healthcare) in 50 mm HEPES (pH 7) according to the manufacturer's instructions. The labeling degree for Alexa680‐RBD was determined based on the monitored absorbance at 280 and 680 nm. Similarly, conjugation of 0.5 mg streptavidin (SA) (BioSpa, Milano, Italy) with heptadentate ITC‐TEKES‐Eu(III)‐chelate (QRET Technologies), termed Eu(III)‐SA, was performed with a 10‐fold molar excess of Eu(III)‐chelate in a 3 h reaction (RT) in 300 μL. The conjugated product was purified with NAP‐5 columns as well. The Eu‐GTP concentration was determined based on the Eu(III) ion concentration by comparing the observed luminescence signal with a commercial Eu(III) standard. The EuCl_3_ standard and DELFIA enhancement solution (DES) were purchased from PerkinElmer Life and Analytical Sciences [52].

### 2.16 Nucleotide association assays with K‐Ras

In the quenching resonance energy transfer (QRET) nucleotide exchange assay [53, 55], Eu(III)‐GDP association and dissociation kinetics were performed in assay buffer 1 (20 mm HEPES, pH 7.5, 1 mm MgCl_2_, 10 mm NaCl, 0.01% Triton‐X 100, 0.005% γ‐globulins). Eu(III) was excited at 340 nm, and the resulting signal was measured with an 800 μs delay at 615 nm for 400 μs. KRAS (400 nm) was first added in 8 μL and mixed with buffer or 2 μL of DARPin 784_F5 or DARPin E3_5 (2 μm). Eu(III)‐GDP (50 nm), premixed with 55 μm Malachite Green (Sigma‐Aldrich) was then added in 8 μL and the signal was monitored after 5 min of incubation. Eu(III)‐GDP association kinetics were followed for 800 s after SOS1 catalytic domain (SOS^cat^, 564–1048) (5 nm) addition in 2 μL. Eu(III)‐GDP association was stopped by adding 2 μL of GDP (10 μm) in each well. Eu(III)‐GDP dissociation kinetics were monitored for 800 s after GDP addition. All reactions were performed in triplicates, and reactions without SOS^cat^ or DARPin were used as controls.

To determine an IC_50_, the assay was performed in an endpoint format. For this purpose, assays were performed in assay buffer 2 (20 mm HEPES, pH 7.5, 1 mm MgCl_2_, 10 mm NaCl, 0.01% NP40) with either 10 nm Alexa647‐GDP or Alexa647‐GTP, respectively. KRAS(1–188) (10 nm) and 0–4 μm of each DARPin (784_F5 and E3_5) were first incubated for 10 min before adding Eu(III)‐GDP or Eu(III)‐GTP (5 nm) in complex with MT2 (2.5 μm, QRET Technologies). After 5 min, the starting signals were monitored and SOS^cat^ (5 nm) was added. Association blocking was monitored after 15 min at 620 nm. Data represent mean ± SD (*n* = 3).

### 2.17 K‐Ras interaction monitoring with Raf‐RBD

The ability of the DARPins to block KRAS interaction with the RAF‐RBD was monitored using TR‐FRET readout. Titration of DARPins 784_F5 or E3_5 (negative control) was performed in assay buffer 1 using an endpoint protocol. For this purpose, Alexa647‐GTP (10 nm) association to KRAS (50 nm) was initiated with SOS^cat^ (5 nm) for 10 min. Thereafter, 0–3 μm 784_F5 or E3_5 control DARPin was added, and the reaction was incubated for 10 min. RAF1‐RBD‐biotin (10 nm) and Eu(III)‐SA (2 nm) were then added, and TR‐FRET signals were monitored after 15 min at 665 nm.

Kinetic determination of the DARPins' ability to block KRAS interaction with RAF1‐RBD was also monitored using a TR‐FRET readout. Avi‐KRAS(1–188) (25 nm), GTP (2 μm), and Eu(III)‐SA (5 nm) were first incubated for 15 min with SOS^cat^ (5 nm) to enable GTP‐Ras formation. Thereafter, Alexa680‐RAF1‐RBD (25 nm) was added and kinetics of KRAS interaction with RAF1‐RBD‐Alexa680 were monitored at 730 nm for 330 s before DARPin (2 μm) addition and RAF1‐RBD—Alexa680 dissociation monitoring for 480 s. In both assays, all individual assay components were added in 2 μL volume and the final volume was 10 μL. All reactions were performed in triplicates, and reactions without DARPin and without SOS^cat^ were used as controls.

### 2.18 Xenograft study

2.5 × 10^6^ HCT116 cells, inducibly expressing the DARPins 784_F5 or E3_5 in 20% matrigel, were subcutaneously implanted in the right flank of 6–8 weeks old NSG mice (NOD.Cg‐*Prkdc* ^scid^ *Il2rg* ^tm1Wjl^/SzJ). Mice were distributed to create homogeneous control and treatment cohorts. In study 1, the non‐induced (−Dox) groups included five animals, while the induced (+Dox) groups included seven animals, of which two were sacrificed on day 10 of treatment for histology. Study 2 only included five animals expressing the DARPins 784_F5. Treatments with doxycycline (0.5 mg/mL + 5% sucrose in drinking water) and control (5% sucrose) were initiated when tumor volumes reached ≥ 125 mm^3^. Tumor volumes were monitored by measuring tumor length (*L*) and width (*W*) using a caliper. Tumor volume (*V*) was calculated using the standard formula: *V* = *W* × *W* × *L*/2, where *L* represents the longest dimension and W the perpendicular shorter dimension. All measurements were performed blind by the same trained investigator to minimize variability. Measurements were taken every 3–4 days, starting 1 week post‐inoculation and twice a week while on treatment. Tumors exceeding the ethical limit or showing signs of ulceration triggered humane endpoint criteria, in accordance with institutional animal care guidelines. All animal experimental procedures were performed according to the Federation of European Laboratory Animal Sciences Associations (FELASA). The animal study protocol was approved by the Institutional Review Board (Comité d'éthique CEEA 50) of University of Bordeaux (protocol code APAFIS10089‐2017052408559377, date of approval 31/05/2018).

### 2.19 Histology

Tumor samples derived from mouse implants were fixed in 10% buffered formalin (Sigma), embedded in paraffin, and evaluated by conventional hematoxylin & eosin (H&E) staining. Antibodies used for immunostaining included those raised against phosphorylated Histone 3 (06‐570, Millipore, Burlington, MA, USA).

## 3 Results

### 3.1 Development and screening of RAS‐binding DARPins

To develop Designed Ankyrin Repeat Proteins (DARPins) binding to RAS, ribosome display was performed in a 96‐well high‐throughput format with the KRAS mutant Q61H (1–169) which was biotinylated via a C‐terminal Avi‐tag. The mutant exhibits a strongly impaired ability to hydrolyze GTP [56]. After four rounds of selection from the DARPin library, *E. coli* was transformed with the selected pool of DARPins and individual clones were picked. *E. coli* crude extracts containing expressed DARPins were prepared using these clones. Binding of DARPins to RAS immobilized via its Avi‐tag was confirmed by ELISA and subsequently corroborated via homogeneous time‐resolved fluorescence (HTRF) for a subset of clones. We then evaluated a set of RAS‐binding DARPins for their ability to block the RAS–RAF interaction and found strongly varying abilities to do so in a RAF1‐RBD based IP (Fig. S1). Here we identified the DARPin 784_F5, which was previously reported as RAS0107 without in‐depth characterization [35], as the most promising candidate which is investigated in detail in the present study.

### 3.2 Recognition of all RAS isoforms via the switch I/II region independently of the nucleotide bound

**Fig. 1:**
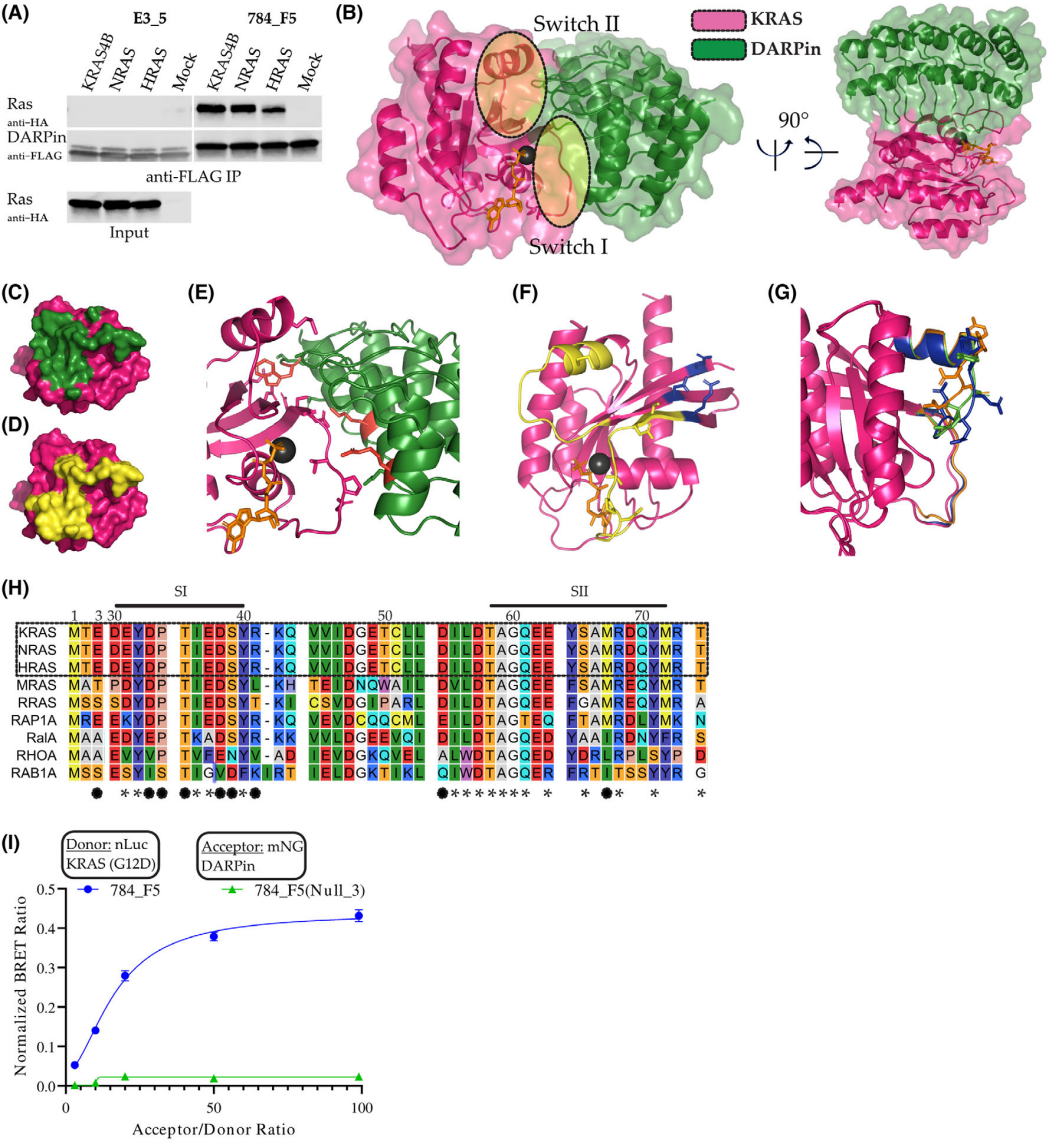
Binding mode of the pan‐RAS DARPin 784_F5. (A) Co‐immunoprecipitation (Co‐IP) of the three RAS isoforms by the DARPin 784_F5. HA‐tagged isoforms were expressed in HEK293T cells and subjected to Co‐IP by FLAG‐tagged DARPins immobilized on anti‐FLAG beads (*n* = 1). (B) Crystal structure of DARPin 784_F5 (green) in complex with KRAS (pink), crystallized in the presence of GMP‐PNP. Two views of the complex are shown, rotated by 90° about a vertical and horizontal axis, with switch 1 and switch 2 regions annotated. (C) Surface representation of KRAS (pink) indicating the area covered when bound to DARPin 784_F5 (green). (D) Surface representation of KRAS (pink) indicating the switch I/II region (yellow). (E) Binding interface of DARPin 784_F5 (green) and KRAS (pink). Residues mediating direct interactions are displayed as sticks. The key residues, W36, K46, and R112, mutated to alanine in 784_F5(Null_3) are colored in red. (F) Residues on KRAS involved in direct and water‐mediated interactions with DARPin 784_F5. Residues located in the switch regions are colored in yellow while those outside are colored in blue. (G) Overlay of the three KRAS molecules within a unit cell. Switch I/II regions are colored by chain, and the residues 61–64 are shown as sticks. (H) Sequence alignment of the switch I/II region of representative small GTPases compared to KRAS. Direct and water‐mediated RAS DARPin interactions are indicated by filled circles below the alignment. Additional interface residues as defined by PDBePISA are indicated by asterisks. (I) BRET assay showing in‐cell target engagement of KRAS(G12D) by the DARPin 784_F5 (*n* = 3, error bars indicate SD). The RAS DARPin interaction was absent for 784_F5(Null_3) carrying W36A, K46A, and R112A mutations. Controls showing comparable expression levels are available in Fig. S4.

To characterize the interaction profile of the DARPin 784_F5 with the individual RAS isoforms in their on‐ and off‐states, we determined its affinity for NRAS, KRAS, and the KRAS mutant G12V using surface plasmon resonance (SPR). Affinities were measured both against the inactive, GDP‐loaded form and the active GTPγS‐loaded form. The DARPin recognized all tested RAS proteins independent of the nucleotide bound. Using a two‐step model, all SPR curves could be fitted well (Fig. S2) and showed highly similar overall *K*_D_ values ranging from 6.7 to 23.8 nM (Table 1). In addition to the affinity measurements, we conducted immunoprecipitation of HA‐tagged RAS isoforms from cell lysate prepared from transfected HEK293T cells. The RAS binder 784_F5 clearly recognizes all isoforms as full‐length proteins also when produced and processed in a mammalian cell (Fig. 1A), while this is not the case for the non‐binding DARPin E3_5, which was used as a control.

**Table 1:** Determination of DARPin 784_F5 affinity. *K*_D_ on KRAS(wt), KRAS(G12V) and NRAS(wt) as measured by surface plasmon resonance (SPR) and fitted with a two‐state model (see Section 2). Individual sensorgrams are displayed in Fig. S2.

	*k*_a1_	*k*_d1_	*K*_D_	*k*_2_	*k*_−2_
	m ^−1^·s^−1^	s^−1^	m	s^−1^	s^−1^
KRAS(wt) – GTPγS	7.53·10^5^	5.12·10^−3^	6.80·10^−9^	1.88·10^−3^	9.45·10^−4^
KRAS(G12V) – GTPγS	6.25·10^5^	5.94·10^−3^	9.51·10^−9^	1.78·10^−3^	9.15·10^−4^
NRAS(wt) – GTPγS	4.52·10^5^	4.68·10^−3^	1.04·10^−8^	2.05·10^−3^	8.74·10^−4^
KRAS(wt) – GDP	6.39·10^5^	4.27·10^−3^	6.69·10^−9^	1.60·10^−3^	9.17·10^−4^
KRAS(G12V) – GDP	3.03·10^5^	7.20·10^−3^	2.38·10^−8^	1.69·10^−3^	1.15·10^−3^
NRAS(wt) – GDP	4.03·10^5^	4.15·10^−3^	1.03·10^−8^	2.12·10^−3^	7.56·10^−4^

We subsequently crystallized the DARPin 784_F5 in complex with KRAS bound to the GTP analog GMP‐PNP to rationalize the interaction profile we observed (Table S2). The DARPin covers the switch I region completely and occupies large areas of the switch II area, mediating direct contacts with both regions, thereby occupying the binding site of the various RAS downstream effectors (Fig. 1B–E). Interestingly, the switch I/switch II regions do not closely resemble known conformations of inactive (GDP‐bound) or active (GTP‐bound) KRAS structures but in both cases adopt an intermediate conformation [57, 58]. While the α2 helix is found in an open conformation similar to the GDP state, the switch II loop aligns more closely to the protein core as observed in other structures [59]. In contrast, the switch I region is further extended from the G‐domain core than expected from reported structures of effector‐ or unbound KRAS, but does not align with the completely opened switch I conformation observed in complex with the RAS GEF SOS1 [59, 60, 61].

Importantly, the structure allows us to rationalize how the DARPin 784_F5 is able to bind RAS in both nucleotide states, even though its epitope—the switch regions—adopts distinct conformations when GDP or GTP is bound. First, three of its direct interactions with KRAS are located on the conformationally rigid β‐sheet (β1‐3), providing a constant region of contact (Fig. 1F). In addition, DARPin 784_F5 shows a high tolerance towards the conformation of the switch II loop. Within the crystal, one unit cell is composed of three KRAS DARPin pairs that, when superimposed, show a surprising variability in the residues 61–64 of KRAS switch II (Fig. 1G). Analyzing the direct and water‐mediated interactions between both molecules reveals partially varying interactions, depending on the conformation of the switch II loop. For example, K134 of DARPin 784_F5 can interact with either E62 or E63 (Fig. S3). These observations align well with the two‐step model used to fit the kinetic binding data. The model is assuming a low‐affinity initial encounter that is followed by a conformational transition to a high‐affinity complex. Here, the initial encounter would likely involve interactions with the β1‐3 sheet and the switch I region, while stable interactions with the highly flexible switch II loop are established in a subsequent step.

Besides its conformational tolerance with regard to the nucleotide state of RAS, the structure suggests shared specificity for the three RAS isoforms KRAS, NRAS, and HRAS. In contrast, an alignment of representative proteins from the RAS and other small GTPases families shows differing residues in at least one direct interaction (hydrogen bond or salt bridge) and two or more further interactions compared to KRAS (Fig. 1H).

To validate the specificity of the DARPin's interaction with KRAS and its ability to engage its target within mammalian cells, we probed the interaction in a BRET assay. While DARPin 784_F5 readily engaged KRAS (G12D) in HEK293T cells, mutating three residues involved in direct interactions (W36A, K46A, and R112A) abolished the interaction (Fig. 1I).

### 3.3 DARPin binding affects SOS‐mediated nucleotide exchange and RAS‐effector engagement

**Fig. 2:**
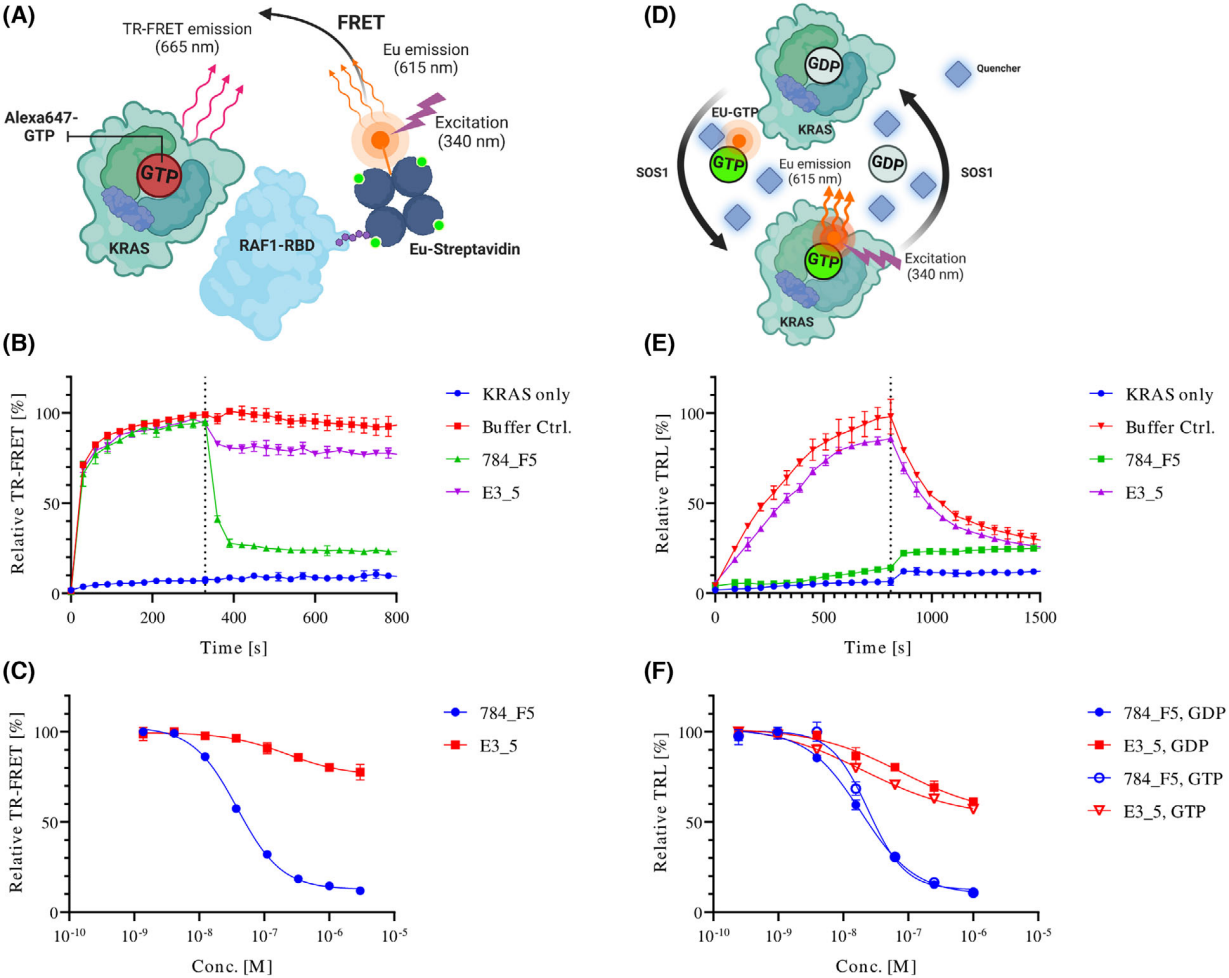
Activity of DARPin 784_F5 on RAS–RAF interaction and SOS‐mediated nucleotide exchange. (A) Schematic representation of the TR‐FRET‐based RAS–RAF assay. The interaction is measured as a FRET signal resulting from Alexa680‐GTP loaded KRAS as acceptor, being bound by the biotinylated RAF1‐RBD in the presence of the donor Eu(III)‐labeled Streptavidin. (B) The RAS–RAF interaction is initiated by SOS‐mediated activation of KRAS4B. As shown in (A), once a stable signal is established, competitors are added and the signal is followed over time. The addition of competitors, or the same amount of buffer is indicated by a vertical broken line. (C) Dose‐inhibition curves of the anti‐RAS DARPin 784_F5 and the non‐binding control DARPin E3_5 in the RAS–RAF interaction assay. (D) Schematic representation of the QRET assay, which allows observation of SOS‐mediated nucleotide exchange. The signal results from SOS‐mediated loading of KRAS with Eu(III)‐labeled GDP or GTP. The Eu signal is quenched while in solution by a soluble quencher. (E) All components are mixed before nucleotide loading is initiated by the addition of SOS and observed over time. Unlabeled GDP is added in excess after 800 s and allows monitoring the back‐exchange of the labeled nucleotide. The signal is measured as time‐resolved luminescence (TRL). (F) Dose‐inhibition curves of the anti‐RAS DARPin 784_F5 and the non‐binding control DARPin E3_5 in the nucleotide exchange assay. Data represent three technical replicates; error bars indicate SD.

Since DARPin 784_F5 binds to the effector lobe of KRAS with high affinity in both nucleotide states, we hypothesized that it would shield KRAS from interacting with its nucleotide exchange effector SOS as well as its effectors such as RAF. Both interactions are essential for the (re)activation of RAS and signal transduction to the MAPK and PI3K/AKT/mTOR pathways. To validate our assumptions, we first investigated the ability of the anti‐RAS DARPin 784_F5 to interfere with the interaction of KRAS with the RAS‐binding domain (RBD) of RAF1 upon GTP loading of KRAS by SOS in a time‐resolved (TR)‐FRET assay (Fig. 2A) [53]. Importantly, the DARPin 784_F5 not only blocked the association of the RAF1‐RBD but also competed with it in a pre‐assembled complex with KRAS while the non‐binding control DARPin E3_5 did not (Fig. 2B). We observed an IC_50_ of 38.44 nm for inhibiting the KRAS‐RAF1 interaction (Fig. 2C). As RAF1 is considered the effector with the highest affinity for KRAS, it is likely that DARPin 784_F5 competes with other effectors, such as PI3K, in a similar manner [62].

We additionally probed SOS‐mediated nucleotide exchange in a quenching resonance energy transfer (QRET) assay (Fig. 2D) [52]. DARPin 784_F5 potently inhibited nucleotide exchange of GDP‐bound KRAS with Eu‐labeled GDP and GTP with IC_50_ values of 18.78 and 34.32 nm, respectively (Fig. 2E,F). In both cases, the observed IC_50_ values adequately reflect the potency expected from the measured *K*_D_. The ability to block both SOS‐mediated nucleotide exchange and the interaction with the RAF1‐RBD underscores the engagement of RAS in both its inactive GDP‐loaded state and the active GTP‐loaded state.

### 3.4 DARPin binding affects KRAS multimerization and supports an asymmetric KRAS dimer model

**Fig. 3:**
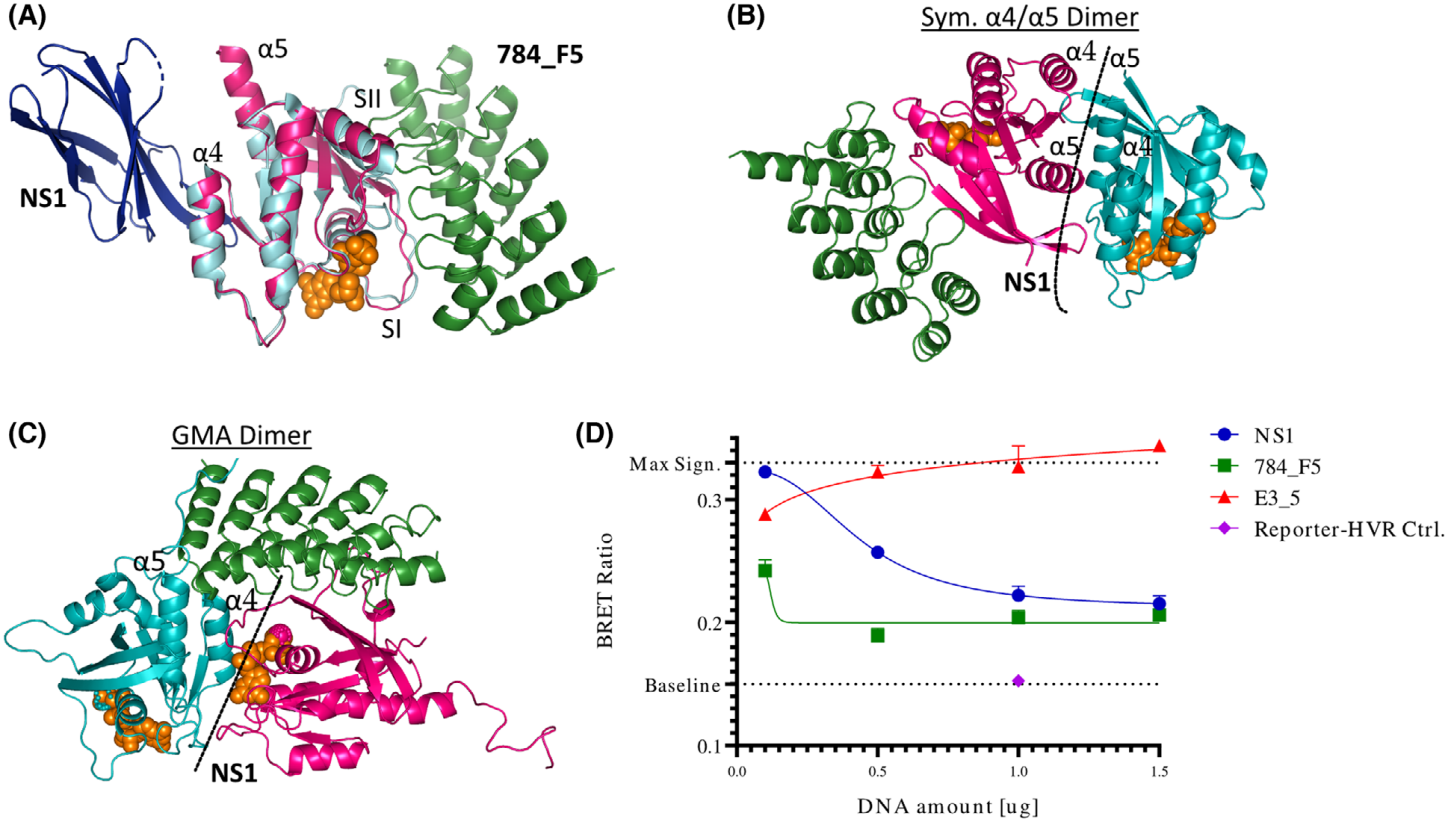
Effect of RAS binders on KRAS nanoclustering. (A) Overlay of DARPin 784_F5 (green) bound KRAS (pink) and NS1 monobody (blue) bound HRAS (cyan) (PDB:5E95). It can be seen that they bind to opposite sides of KRAS. (B) Overlay of DARPin 784_F5 (green) bound to KRAS (pink) and the symmetric α4‐α5 dimer model. The binding site of the monobody NS1 is indicated by a dashed line. (C) Steric interference of the DARPin 784_F5 with an asymmetric dimer interface. The binding site of the monobody NS1 is indicated by a dashed line. (D) KRAS nanoclustering measured by BRET^2^ (*n* = 3, error bars indicate SD). KRAS was N‐terminally fused to either nanoLuciferase (Donor) or mNeonGreen (Acceptor) and co‐transfected with the indicated inhibitors and controls in HEK293T cells. The signal observed for the non‐binding DARPin E3_5 represents the maximum BRET signal and accounts for general effects of transfection and DARPin expression. The baseline signal is defined by the non‐specific interaction of the reporters anchored in the membrane via the KRAS4B‐HVR sequence.

In the MAPK pathway, multiple RAS proteins assemble at the cell membrane with RAS‐effector proteins from the RAF family to enable downstream signal transduction [63, 64, 65, 66]. Despite increasing evidence for a structurally defined RAS self‐association, the exact interaction interface(s) and size of such multimers remain a matter of ongoing investigation and discussion [67, 68, 69]. For testing the impact of RAS binders on KRAS nanoclustering, we consider two models as particularly relevant that were only reported for the GTP‐bound state of KRAS: a symmetric α4/α5 dimer as well as a GTP‐mediated asymmetric (GMA) dimer model that involves the switch I region in addition to the α4/α5 helices [28, 70, 71]. We hypothesized that blocking one of the involved epitopes at a time, the α4/α5 helices or the switch I region, would allow to investigate the relevance of the individual epitope in the formation of RAS nanoclusters. To do this, we utilized the previously reported NS1 monobody [28] recognizing the α4‐β6‐α5 region of the allosteric lobe and the here reported DARPin 784_F5, recognizing the switch I/II effector lobe (Fig. 3A). The alignment of the complexes of DARPin 784_F5‐ or monobody NS1‐bound RAS with both dimer models of KRAS clearly shows that the NS1 monobody would interfere with both models, while the DARPin 784_F5 would only be able to interfere with the asymmetric GMA dimer model (Fig. 3B,C).

We utilized a modified BRET^2^ assay, previously validated in the context of RAS nanoclustering [70], to assess the impact of these binders. Briefly, KRAS was N‐terminally fused to either nanoLuciferase, serving as the BRET donor upon addition of its substrate Coelenterazine 400a, or mNeonGreen, serving as the BRET acceptor when both proteins are in close proximity. We showed that neither HVR‐truncated KRAS4B (1–169) nor the reporters fused to the KRAS4B‐HVR yield a productive BRET^2^ signal (Fig. S5). This highlights that KRAS‐like membrane localization is insufficient to induce cluster‐like local concentration gradients while indicating that the KRAS G‐domain is incapable of forming nanoclusters without membrane attachment. This supports an ordered assembly of KRAS4B at the membrane.

For both RAS‐targeted binders, 784_F5 and NS1, we observed a dose‐dependent inhibition of KRAS nanoclustering to similar levels in the BRET^2^ assay (Fig. 3D). As monobody NS1 and DARPin 784_F5 recognize two distinct, non‐overlapping epitopes, these results indicate the relevance of both the DARPin 784_5‐targeted effector and the allosteric lobe, recognized by the monobody NS1, for KRAS nanoclustering.

Notably, the DARPin 784_F5 could also mediate this effect via the inhibition of nucleotide exchange, assuming that KRAS nanoclustering requires preceding SOS‐mediated activation. Unfortunately, the overlapping epitopes of the RAF‐RBD and the DARPin 784_F5 do not allow probing the amount of GTP‐loaded KRAS in a cellular environment by RAF‐RBD immunoprecipitation. Consequently, we cannot conclude whether steric occlusion of the effector lobe or the enrichment of GDP‐KRAS is responsible for the impairment of nanoclustering.

### 3.5 Intracellular anti‐RAS DARPin expression affects proliferation of RAS‐dependent cell lines in 2D & 3D cultures

**Fig. 4:**
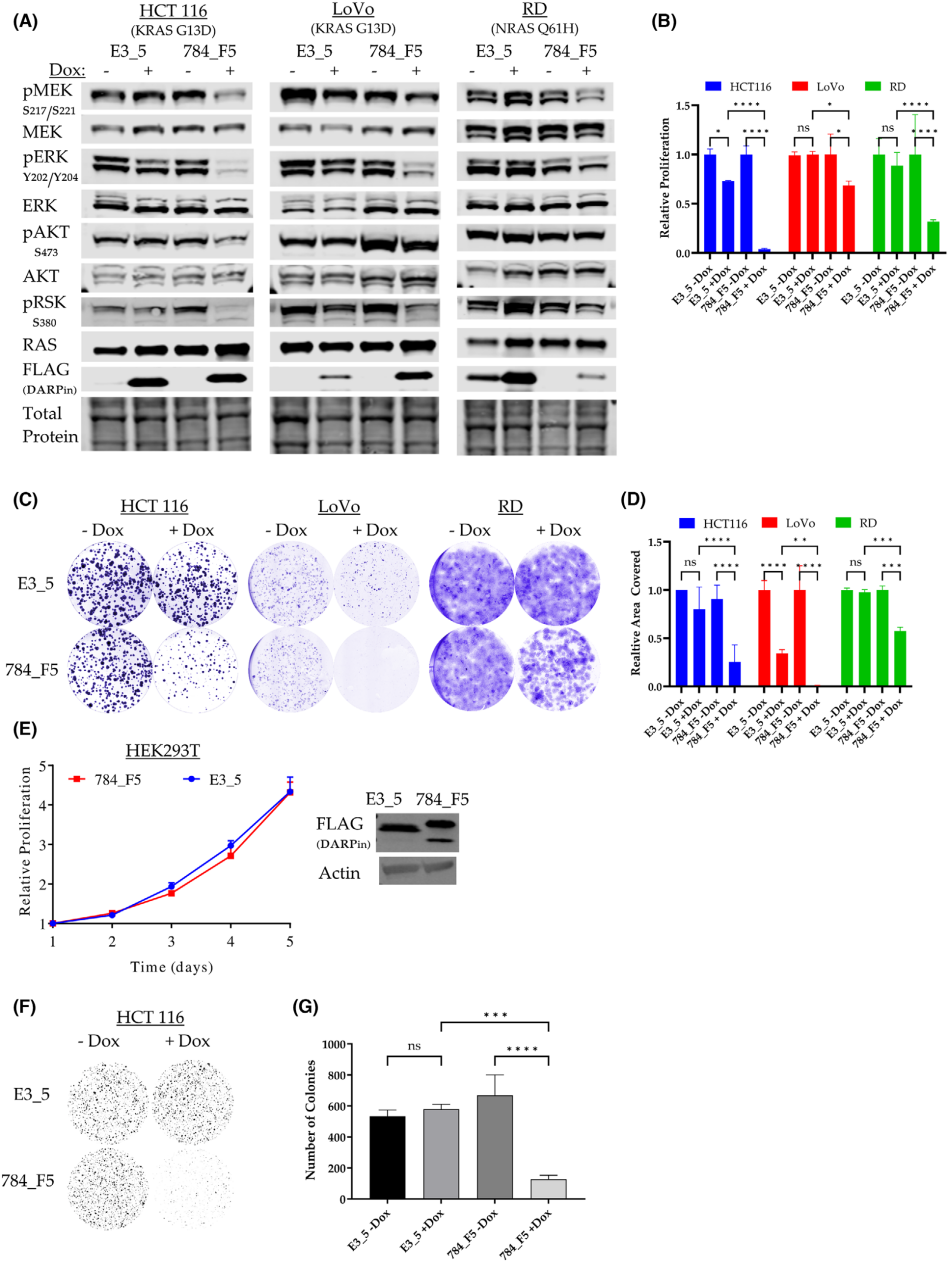
Inhibition of DARPin‐mediated RAS‐signaling limits proliferation of RAS‐dependent cell lines (A) Effect of the DARPin 784_F5 on RAS downstream signaling. RAS‐dependent cell lines stably expressing the anti‐RAS DARPin 784_F5 or the non‐binding DARPin E3_5 in a doxycycline‐inducible manner were subjected to western blotting 24 h post‐induction (*n* = 1). (B) Proliferation of indicated cell lines in the presence and absence of anti‐RAS or control DARPins over 4 days in 2D cultures (*n* = 3, error bars indicate SD). (C) 2D – Colony formation assay. Representative images of cells stained with crystal violet after 10 days of growth. (D) Quantification and statistical evaluation of differences in colony formation (*n* = 3, error bars indicate SD). (E) Proliferation of HEK293T cells, stably expressing the anti‐RAS DARPin 784_F5 or the control DARPin E3_5 as shown by western blot (*n* = 3, error bars indicate SD). The double band observed for DARPin 784_F5 corresponds to (un)folded fraction of the DARPin on the SDS gel due to its high stability. (F) 3D – Soft agar assay. Representative images of stained samples after 10 days of growth. (G) Quantification and statistical evaluation of differences in anchorage‐independent growth (*n* = 3, error bars indicate SD). All experiments were performed as three biological replicates. Statistical significance was calculated via two‐way ANOVA. Significance levels are represented in the following nomenclature: *P ≤ 0.05; **P ≤ 0.01; ***P ≤ 0.001; ****P ≤ 0.0001.

In the next step, we investigated whether DARPin 784_F5 would affect RAS downstream signaling and proliferation in RAS‐dependent cell lines [72]. We hypothesized that interference with nucleotide exchange and downstream effector engagement would result in the inhibition of RAS downstream signaling pathways. To test this, we stably transduced the RAS‐dependent cell lines HCT116 (KRAS G13D), LoVo (KRAS G13D), and RD (NRAS Q61H) with doxycycline‐inducible anti‐RAS DARPin 784_F5 or the non‐binding DARPin E3_5. DARPin expression was induced with doxycycline for 24 h, and the activity of the two most relevant RAS‐mediated signaling pathways, MAPK/ERK and PI3K/AKT, was determined by western blot analysis using phospho‐specific antibodies. The expression of the control DARPin E3_5 did not show an effect when compared to non‐induced cells (Fig. 4A). In contrast, the levels of phospho‐MEK (S217/S221), phospho‐ERK (T202/Y204), and phospho‐RSK (S380) were strongly reduced in all three cell lines 24 h after induction of DARPin 784_F5 as compared to non‐induced cells or cells expressing the control DARPin E3_5, indicating inhibition of the MAPK/ERK pathway. In addition, the reduction of phospho‐AKT (S473) upon expression of DARPin 784_F5 was evident in the colon carcinoma cell lines HCT116 and LoVo, showing that also the PI3K/AKT pathway can be impaired by DARPin 784_F5. The unaffected AKT phosphorylation in the rhabdomyosarcoma cell line RD is likely due to cancer type‐specific engagement of RAS downstream pathways, as, for example, previously demonstrated for lung versus pancreatic tumors [73].

Based on the successful downregulation of KRAS signaling through its downstream pathways, it can be expected that the anti‐RAS DARPin 784_F5 should have an impact on proliferation in RAS‐dependent cell lines. As shown in Fig. 4B–D, cell proliferation and colony formation in 2D cultures were significantly reduced upon the expression of the anti‐KRAS DARPin 784_F5 in all tested cell lines. A minor decrease in proliferation and colony formation was also observed upon the induction of the control DARPin E3_5 in the cell lines HCT116 and LoVo. In these cases, doxycycline and the strong overexpression of an irrelevant protein might have caused this effect.

To ensure that the DARPin 784_F5 does not possess general cytotoxicity, its effect on HEK293T cells lacking mutated RAS was tested. HEK293T cells were therefore transduced with DARPin‐encoding lentivirus, and the proliferation of selected cells expressing either the anti‐RAS DARPin 784_F5 or the control DARPin E3_5 was analyzed. No difference in cell proliferation was observed when comparing control and anti‐RAS DARPin (Fig. 4E). These results indicated that DARPin‐mediated inhibition of RAS is not generally toxic to cells.

In addition to cell proliferation, we subsequently investigated whether expression of the anti‐KRAS DARPin 784_F5 also affects anchorage‐independent growth of cancer cells. For this 3D assay, we chose the HCT116 cell line to compare it to the results reported for other RAS‐targeted affinity reagents, such as the DARPins K27 and K55, as well as the single domain antibody iDab6 [30, 74]. We found that anchorage‐independent growth was strongly reduced upon the expression of the anti‐KRAS DARPin 784_F5 compared to all other conditions (Fig. 4F,G).

Taken together, all of the performed assays confirmed a strong reduction in proliferation and colony formation in response to the expression of DARPin 784_F5 in the tested cell lines, reflecting its potential for suppressing tumor formation.

### 3.6 Intracellular anti‐RAS DARPin expression allows tumor control *in vivo*

**Fig. 5:**
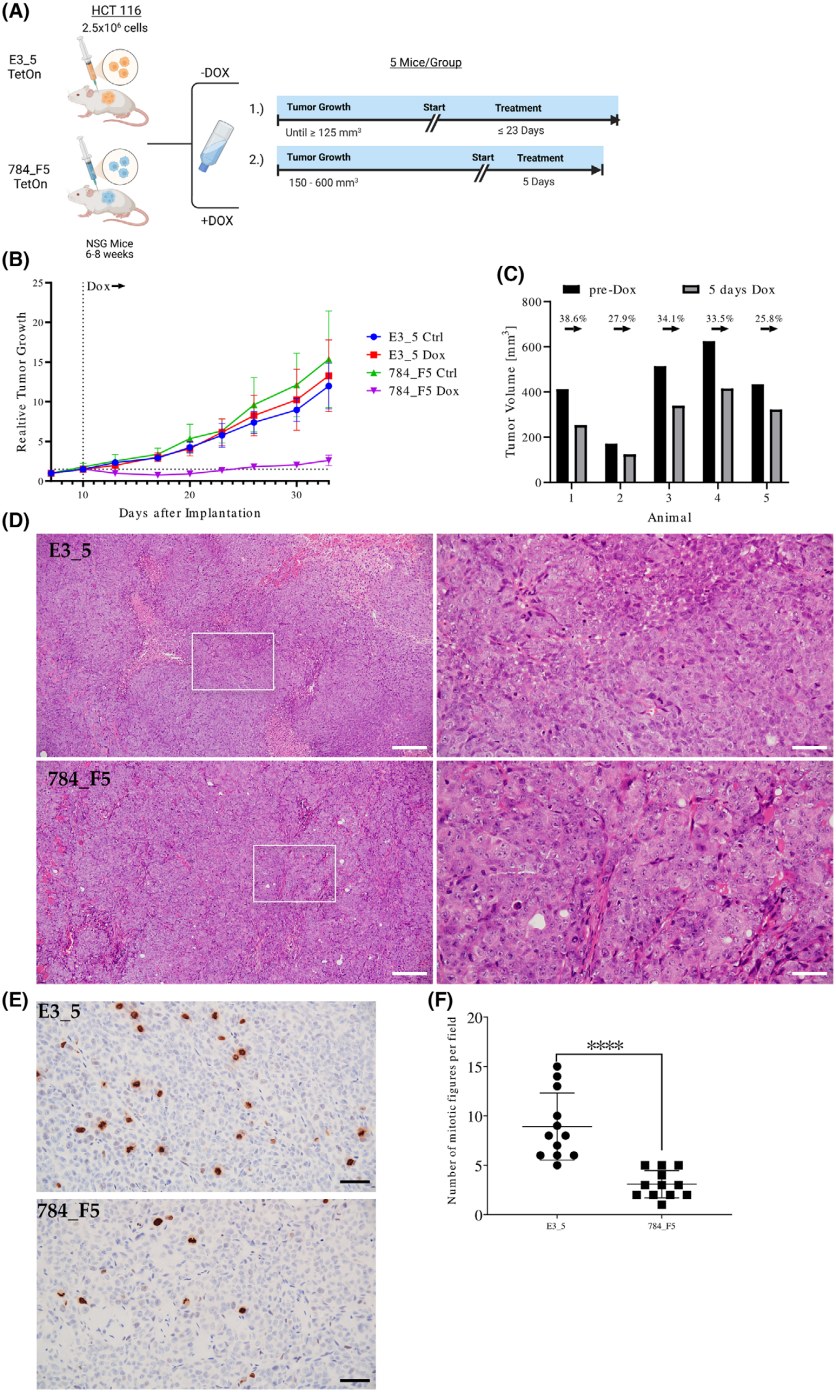
The pan‐RAS DARPin 784_F5 suppresses tumor growth *in vivo*. (A) Schematic representation of the study design. Mice were divided into two groups per cell line (HCT116 Tet On 784_F5/E3_5) and one of which was fed doxycycline by drinking water (2 mg Dox·mL^−1^). In study one, smaller tumors were followed up for up to 23 days while in study two, tumors were allowed to grow larger before the start of treatment. (B) Relative tumor growth as observed in study one was calculated based on the tumor size on day 7 post‐implantation. The average tumor size ± standard deviation at the time point of doxycycline‐mediated induction of DARPin expression is indicated as a dotted line. The individual tumor volumes are displayed in Fig. S6A. (C) Tumor volumes of five individual animals as observed in study two before and after 5 days of doxycycline treatment. The relative changes in tumor volume are indicated. (D) Representative hematoxylin/eosin stainings from subcutaneous HCT116 xenografts obtained after 10 days on doxycycline treatment during study one. Insets indicate the area represented at higher magnification (right panels). Scale bars: 200 μm. (E) Representative immunostainings for phosphorylated histone H3 (Ser10) as a mitotic marker in xenograft sections as described in (D). Scale bar: 200 μm. (F) Quantification of mitotic figures positive for phosphorylated Ser10‐histone H3 in randomly selected fields. Two independent xenografts per condition were used (*n* = 6 fields were quantified per section, error bars indicate SD). Statistical significance was calculated via Student's *t*‐test. Significance levels are represented in the following nomenclature: *P ≤ 0.05; **P ≤ 0.01; ***P ≤ 0.001; ****P ≤ 0.0001.

Exploring the effect of the anti‐RAS DARPin 784_F5 in a xenograft model was the consequent next step based on its potency in the *in vitro* assays. We used the pre‐established, inducible HCT116 cell lines expressing either the non‐binding DARPin E3_5 or the anti‐RAS DARPin 784_F5 upon exposure to doxycycline to establish tumors in NSG mice. Importantly, we allowed tumor formation to a volume of at least 125 mm^3^ before treatment (Fig. 5A). Addition of doxycycline to the drinking water resulted in a 27–66% (50% average) reduction in tumor burden within the first week of treatment for the group expressing DARPin 784_F5, while the control groups doubled their tumor volume on average (Fig. 5B). A subset of mice was sacrificed around the time of maximal response (day 10 on doxycycline) and processed for histopathological characterization. Tumors expressing DARPin 784_F5 displayed reduced nuclear pleomorphism, increased stromal infiltration, and reduced proliferation as assessed by phosphorylated histone H3 (Ser10), when compared to the control DARPin E3_5 (Fig. 5D–F).

However, we observed a slow but steady increase in tumor volume in the DARPin 784_F5‐treated group after the first week, likely resulting from selective pressure leading to a loss of DARPin 784_F5 expression (Fig. S6b). To confirm the tumor regression seen in the initial study, we tested the effect of DARPin 784_F5 in mice bearing much larger tumors (150–600 mm^3^) and observed a similar reduction in tumor volume within 5 days of treatment (Fig. 5C). To our knowledge, this is the first report of tumor regression and/or control of larger tumors in response to the intracellular expression of a RAS‐directed protein binder [27, 74, 75].

## 4 Discussion

We isolated the DARPin 784_F5 that does not distinguish between RAS isoforms or mutants, as shown by surface plasmon resonance (SPR) and co‐immunoprecipitation (Co‐IP). Remarkably, we also could not find a significant preference for the RAS‐on/RAS‐off states represented by GDP or GTPγS‐bound KRAS. The ability to recognize the switch I/II epitope similar to the binding sites of RAS downstream effectors, demonstrated by x‐ray crystallography, is rather surprising, considering the conformational flexibility of this region. While the natural RAS‐binding domains of RAF are highly dependent on the RAS‐GTP conformation of mainly the switch I region, engineered variants of the RAF1‐RBD were shown to bind independently of the nucleotide bound [76]. However, both the two‐step binding model necessary to fit the binding kinetics of the DARPin 784_F5 to KRAS and the adaptive binding interface observed within the complex are in line with the compatibility for both nucleotide states. In biochemical assays, the DARPin was characterized as a dual inhibitor of SOS‐mediated nucleotide exchange and interaction of RAS with the RAF1‐RBD with IC_50_ of 34.3 and 38.4 nm respectively.

Further, the availability of affinity reagents binding to distinct epitopes on KRAS allowed us to investigate their involvement in KRAS nanoclustering. We chose the non‐overlapping RAS binders 784_F5 and NS1 to assess the effect of blocking either the α4/α5 allosteric lobe or the switch I/II effector lobe. By considering the blocked epitope, we could validate whether both interfaces are critical for KRAS dimer formation. Our results demonstrate that the switch I/II binder 784_F5 interferes with BRET^2^‐monitored nanoclustering at least to the same degree as the α4/α5‐targeted NS1 monobody, supporting the relevance of both interfaces for RAS nanoclustering. These observations are consistent with recent findings that describe the involvement of both the allosteric and the effector lobes in the GTP‐dependent formation of KRAS dimers and higher‐order oligomers [70, 71]. In particular, Lee and Lee have provided biochemical evidence for the interaction of both interfaces in the context of nanoclustering in an NMR‐based approach, by establishing a system in which up to four KRAS molecules, labeled at two epitopes, can freely diffuse within nanodiscs [71]. We are confident that RAS affinity reagents such as the here described DARPin are valuable tools in validating the epitopes involved in RAS nanoclustering as they allow for a well‐defined occlusion of a specific epitope on RAS. This enables researchers to elucidate the complex mechanisms driving RAS nanoclustering and its role in signal transduction, ultimately paving the way for the development of novel therapeutic strategies targeting RAS‐driven cancers.

Recently, the development of covalent small‐molecule inhibitors for KRAS (G12C), targeting either its inactive, GDP state, or active, GTP state, led to the discussion of whether one option provides a significant advantage over the other [77, 78, 79]. Additional research, based on the KRAS (G12D) inhibitor MRTX 1133 [13] and the KRAS (G12C) inhibitor BBO‐8520 (NCT06343402) explores the consequences of recognizing both states. While Guillard et al. [30] developed DARPins specific for each one of those states, the DARPin 784_F5 is the only nucleotide‐independent binder recognizing the RAS effector lobe that was functionally characterized in cells [22]. In addition, reports on two other well‐characterized RAS affinity reagents, the GTP‐RAS specific iDab6 and the allosteric NS1 monobody, provide results from xenograft models [74, 75]. In these case studies, the RAS affinity reagents can only delay or control tumor progression but do not cause tumor regression, as observed for the DARPin 784_F5. Notably, the scFv iDab6, in an enhanced, membrane‐targeted version, was tested in the same HCT116 model. Whether these effects are indeed determined by the specificity for both GDP‐bound and GTP‐bound RAS and thus by the exact mechanisms of inhibition described here, or are resulting from individually differing expression levels, remains to be shown. Taken together, the anti‐RAS DARPin 784_F5 described here completes a set of RAS binders that originate from the same protein scaffold 32, recognize highly similar epitopes with comparable affinities, but recognize RAS either in its GDP state (K27), GTP state (K55) or both (784_F5). This might present a unique opportunity to probe the consequences of (pan)‐RAS inhibition based on state‐dependent target engagement. Furthermore, recent advances in the technology of lipid nanoparticles (LNPs) could hold promise for the delivery of RAS‐targeted biologics *in vivo*, as shown by the lab of Andrew Tsourkas [80]. In particular, the delivery of the pan‐RAS^OFF^ DARPin K27 formulated in LNPs resulted in a reduction of tumor load in a NRAS‐mutant hepatocellular carcinoma model.

## 5 Conclusions

With this study, we add the pan‐RAS, nucleotide‐independent, switch I/II‐targeting DARPin 784_F5 to the collection of anti‐RAS biologics. This allowed us to link mechanisms of RAS inhibition and their consequences on RAS downstream signaling, proliferation, and *in vivo* growth of RAS‐dependent cell lines. The expanding library of macromolecular tool compounds allows us to identify and validate important concepts of RAS‐associated vulnerabilities bypassing the limitations of small‐molecule generation. The nucleotide‐independent binding of 784_F5 to RAS contributes to ongoing discussions about the most beneficial conformational state of RAS to target for therapeutic interventions. Our data suggest that inhibiting both the active and inactive states of RAS can lead to potent anti‐tumor effects. However, further studies are needed to elucidate which level of RAS inhibition can provide a reasonable therapeutic window. Taken together, our findings highlight the importance of understanding the complexities of RAS biology in the quest for effective cancer treatments.

## Conflict of interest

AP is a cofounder and shareholder of Molecular Partners AG who is commercializing the DARPin technology. The other authors declare no conflict of interest.

## Author contributions

Conceptualization, AP, WV, JNK, and JVS; investigation, JNK, WV, KK, GN‐D, RT, MS, RD, PE, DS, ER, MJN; resources, AP, KK, DS, and CS; writing – original draft preparation, JNK and WV; writing – review and editing, JNK, AP, and WV; visualization, JNK; supervision, AP and CS; project administration, AP; funding acquisition, AP. All authors have read and agreed to the published version of the manuscript.

## Peer review

The peer review history for this article is available at https://www.webofscience.com/api/gateway/wos/peer‐review/10.1002/1878‐0261.70061.

## Supporting information


**Fig. S1.** RAF1‐RBD‐based immunoprecipitation.
**Fig. S2.** Surface plasmon resonance (SPR) shows binding of DARPin 784_F5 to KRAS (wt), KRAS (G12V) and NRAS loaded with GDP or GTPγS.
**Fig. S3.** Summary of direct and water‐mediated interactions of DARPin 784_F5 in complex with KRAS.
**Fig. S4.** BRET reporter expression levels.
**Fig. S5.** Validation of BRET^2^ reporter assay for KRAS nanoclustering.
**Fig. S6.** HCT116 xenograft.
**Table S1.** Expression constructs.
**Table S2.** Data collection and refinement.

## Data Availability

Coordinates and structure factors have been deposited at the Protein Data Bank (PDB) and are available at PDB‐ID: 9GTK. Other data supporting the findings of this study are available from the corresponding author on reasonable request.
