# Supplementary material for: A nucleotide‐independent, pan‐RAS‐targeted DARPin elicits anti‐tumor activity in a multimodal manner
Source: Mol Oncol. 2025 Jun 15;19(11):3266–86. doi: 10.1002/1878-0261.70061 (PMC12591329; doi:10.1002/1878-0261.70061)
Supplement: Supplementary file 1 — Fig. S1. RAF1‐RBD‐based immunoprecipitation. Fig. S2. Surface plasmon resonance (SPR) shows binding of DARPin 784_F5 to KRAS (wt), KRAS (G12V) and NRAS loaded with GDP or GTPγS. Fig. S3. Summary of direct and water‐mediated interactions of DARPin 784_F5 in complex with KRAS. Fig. S4. BRET reporter expression levels. Fig. S5. Validation of BRET2 reporter assay for KRAS nanoclustering. Fig. S6. HCT116 xenograft. Table S1. Expression constructs. Table S2. Data collection and refinement. [file MOL2-19-3266-s001.pdf]

Supplementary Materials

for

A nucleotide-independent, pan-RAS-targeted DARPin elicits anti-tumor activity in a multimodal manner

Authors

Jonas N. Kapp[1], Wouter P. R. Verdurmen[1], Jonas V. Schaefer[1], Kari Kopra[2], Gabriela Nagy-Davidescu[1], Elodie Richard[3], Marie-Julie Nokin[4,8], Patrick Ernst[1], Rastislav Tamaskovic[1], Martin Schwill[1], Ralph Degen[1], Claudia Scholl[5,6], David Santamaria[7,8], and Andreas Plückthun[1*]

Institutions

1 Department of Biochemistry, University of Zurich, Switzerland
2 Department of Chemistry, University of Turku, Finland
3 Bordeaux Institute of Oncology (BRIC), INSERM U1312, University of Bordeaux, France
4 Laboratory of Biology of Tumor and Development (LBTD), GIGA-Cancer, University of Liege, Belgium
5 Division of Applied Functional Genomics, German Cancer Research Center (DKFZ), Germany
6 National Center for Tumor Diseases (NCT) Heidelberg, Germany
7 Centro de Investigación del Cáncer, CSIC-Universidad de Salamanca, Spain
8 University of Bordeaux, INSERM U1218, ACTION Laboratory, IECB, France

* Corresponding author: plueckthun@bioc.uzh.ch

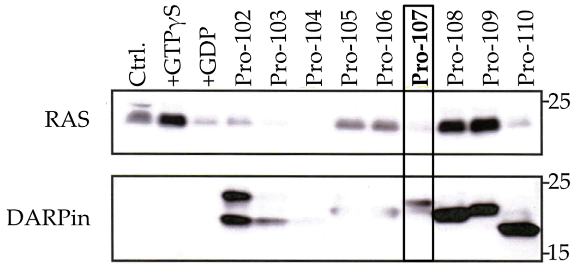

Figure S1. RAF1-RBD based immunoprecipitation. Expression plasmids for nine DARPins selected to bind to KRAS were transfected into HEK293T cells. Active (GTP-loaded) RAS was immunoprecipitated by RAF1-RBD-coated beads. DARPins competing with the RAS-RAF interface and/or specific for GDP-RAS should not be co-precipitated. The DARPin described in this study is highlighted.

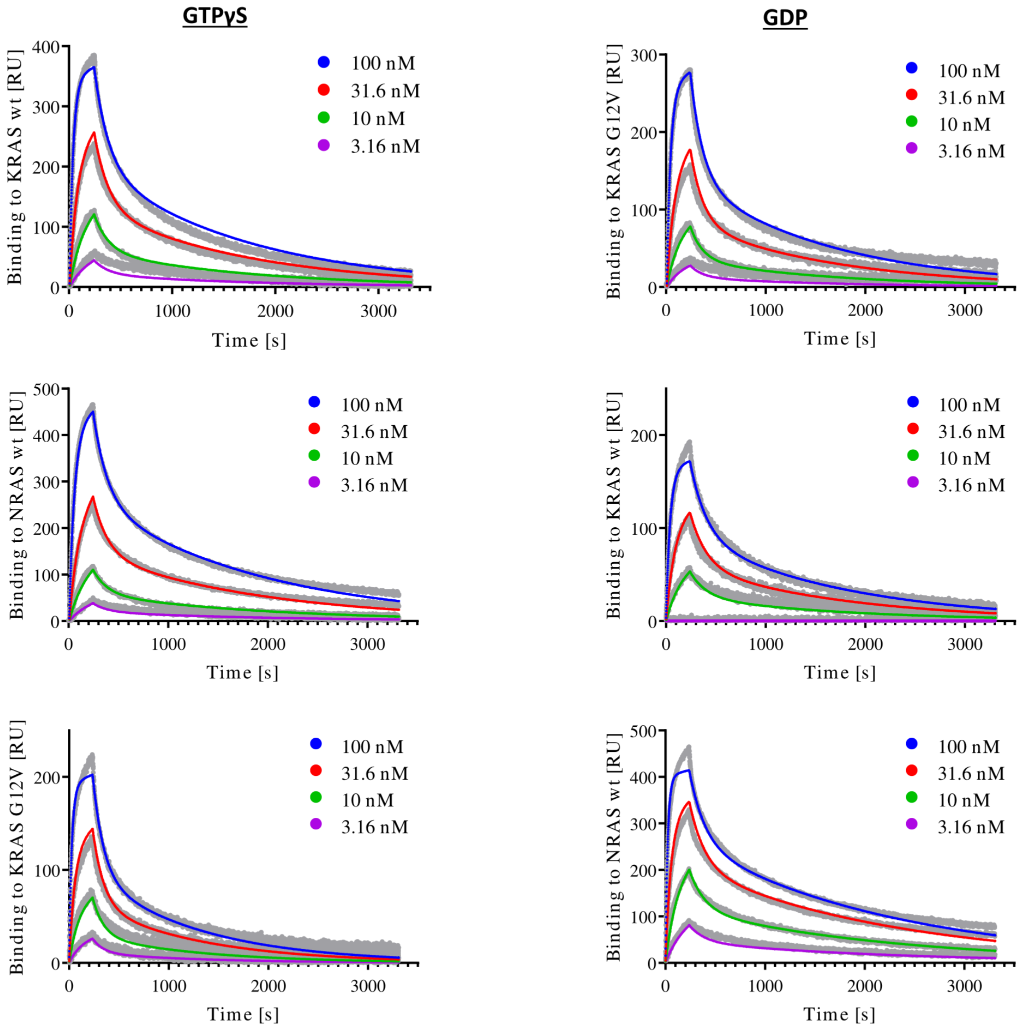

Figure S2. Surface plasmon resonance (SPR) shows binding of DARPin 784_F5 to KRAS (wt), KRAS (G12V) and NRAS loaded with GDP or GTPγS. SPR was used to investigate DARPin binding to the indicated biotinylated RAS variants loaded with different nucleotides, immobilized on a streptavidin biosensor chip. Data points are indicated in grey while the colored lines represent the fitted model.

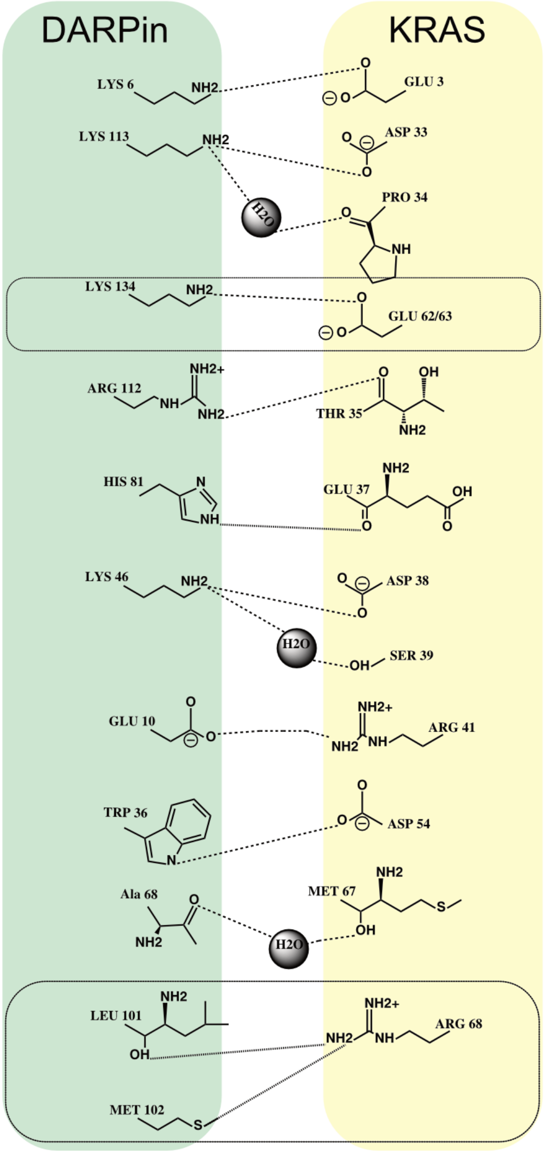

Figure S3. Summary of direct and water-mediated interactions of DARPin 784_F5 in complex with KRAS.

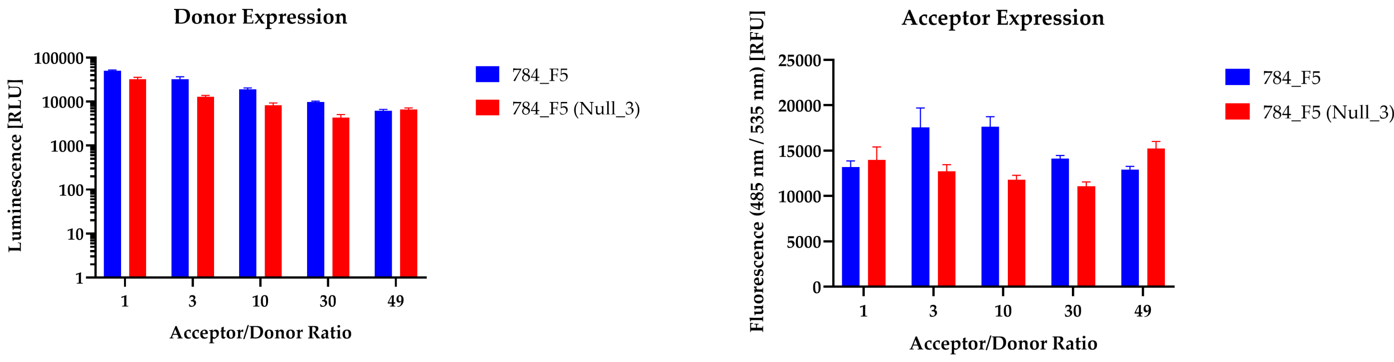

Figure S4. BRET reporter expression levels. Expression levels of nLuc-KRAS(G12D) (donor) and mNeongreen-DARPin (acceptor) at different ratios of transfected donor and acceptor plasmids. This has been tested for both the original 784_F5 and the non-interacting triple mutant (Null_3). It can be seen that the relative reporter expression is comparable for both constructs, showing that the different BRET signal are not a result of differences in expression levels.

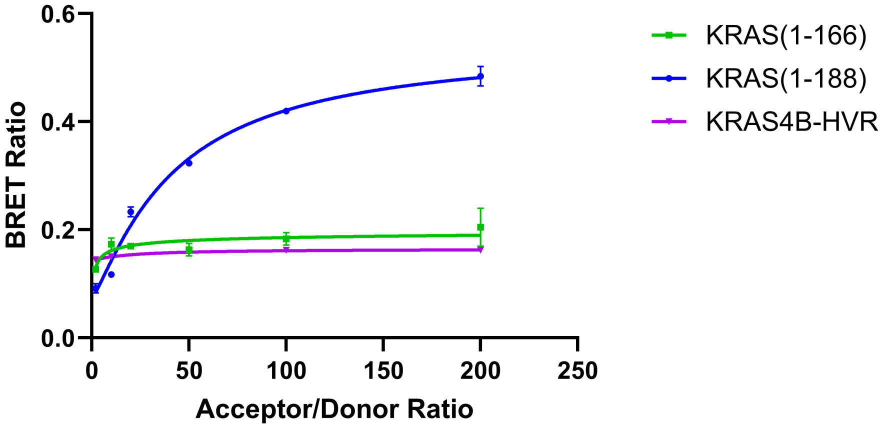

Figure S5. Validation of BRET[2] reporter assay for KRAS nanoclustering. Acceptor/Donor titration of HVR-truncated KRAS(1-166) in green, BRET-reporters fused to the KRAS4B-HVR in violet and full-length KRAS(1-188) in blue. A productive BRET2 signal is only observed for full-length KRAS(1-188).

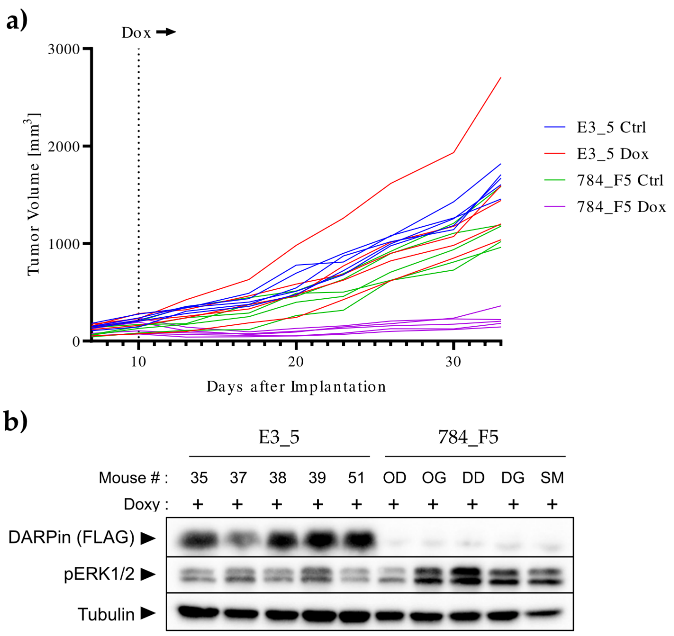

Figure S6. HCT116 xenograft. (a) Tumor volumes of the individual animals from xenograft experiment one. **(b)** Western blot of tumor lysates from animals xenografted with HCT116 cells that either express the control DARPin E3_5 or the anti-RAS DARPin 784_F5. Tumors were harvested at the end of study two, 6 days post-induction of DARPin expression. A strongly reduced DARPin expression is observed for tumors derived from HCT116 cells, expressing the anti-RAS DARPin 784_F5.

Table S1. Expression Constructs

DARPin 784_F5	GSDLGKKLLEAARAGQDDEVRILMANGADVNAEDTWGSTPLHLAAKTGHLEIV<br>EVLLKTGADVNASDAVGHTPLHLAAHKGHLEIVEVLLKTGADVNALDLMGWTP<br>LHLAARKGHLEIVEVLLKHGADVNAQDKFGKTPFDLAIDNGNEDIAEVLQKAA<br>KLN
DARpin E3_5	GSDLGKKLLEAARAGQDDEVRILMANGADVNATDNDGYTPLHLAASNGHLEIV<br>EVLLKNGADVNASDLTGITPLHLAAATGHLEIVEVLLKHGADVNAYDNDGHTP<br>LHLAAKYGHLEIVEVLLKHGADVNAQDKFGKTAFDISIDNGNEDLAEILQKLN
mNeonGreen-X	MVSKGEEDNMASLPATHELHIFGSINGVDFDMVGQGTGNPNDGYEELNLKSTK<br>GDLQFSPWILVPHIGYGFHQYLPYPDGMSPFQAAMVDGSGYQVHRTMQFEDGA<br>SLTVNYRYTYEGSHIKGEAQVKGTGFPADGPVMTNSLTAADWCRSKKTYPNDK<br>TIISTFKWSYTTGNGKRYRSTARTTYTFAKPMAANYLKNQPMYVFRKTELKHS<br>KTELNFKEWQKAFTDVMGMDELYKGGGGSG
NanoLuciferase-X	MVFTLEDFVGDWRQTAGYNLDQVLEQGGVSSLFQNLGVSVTPIQRIVLSGENG<br>LKIDIHVIIPYEGLSGDQMGQIEKIFKVVYPVDDHHFKVILHYGTLVIDGVTP<br>NMIDYFGRPYEGIAVFDGKKITVTGTLWNGNKIIDERLINPDGSLLFRVTING<br>VTGWRLCERILAGGGGSG
KRAS(1-186)-<br>TEV-Avi-10xHis	MTEYKLVVVGAGGVGKSALTIQLIQNHFVDEYDPTIEDSYRKQVVIDGETCLL<br>DILDTAGQEEYSAMRDQYMRTGEGFLCVFAINNTKSFEDIHHYREQIKRVKDS<br>EDVPMVLVGNKCDLPSRTVDTKQAQDLARSYGIPFIETSAKTRQGVDDAFYTL<br>VREIRKHKEKMSKDGKKKKKKSKTKCVGSAENLYFQSGGGLNDIFEAQKIEWH<br>EHHHHHHHHHH

Table S2. Data Collection and Refinement
KRAS in complex with DARPin 784_F5, PDB ID 9GTK

**Resolution range**	47.28 - 2.0 (2.071 - 2.0)
**Space group**	P 21 21 21
**Unit cell**	58.32 152.83 149.15 90 90 90
**Total (Unique) reflections**	90878 (8966)
**Completeness (%)**	100.00
**Wilson B-factor**	44.68
**Reflections used in refinement**	90878 (8964)
**Reflections used for R-free**	4544 (448)
**R-work**	0.1694 (0.3773)
**R-free**	0.2042 (0.3852)
**Number of non-hydrogen atoms**	8911
**macromolecules**	8059
**ligands**	288
**Protein residues**	1011
**RMS(bonds)**	0.026
**RMS(angles)**	2.28
**Ramachandran favored (%)**	98
**Ramachandran allowed (%)**	1.8
**Ramachandran outliers (%)**	0.098
**Rotamer outliers (%)**	1.9
**Clashscore**	4.26
**Average B-factor**	37.10
**macromolecules**	34.21
**ligands**	67.71
**solvent**	62.78
**Number of TLS groups**	6
